# Predicting the efficacy of recombinant human thrombopoietin in treating cancer therapy-related thrombocytopenia: based on stacking ensemble methods

**DOI:** 10.3389/fonc.2026.1751214

**Published:** 2026-05-15

**Authors:** Kun Hou, Rui Huangfu, Zhijuan Guo, Yan Gao, Haiwen Lu, Zhongwu Li, Zhenfei Wang

**Affiliations:** 1 Department of Pharmacy, Peking University Cancer Hospital/Affiliated Cancer Hospital of Inner Mongolia Medical University, Hohhot, Inner Mongolia, China; 2 The Laboratory for Inheritance and Development of Integrated Chinese (Mongolian) and Western Medicine in Anti-Tumor Therapy, Peking University Cancer Hospital/Affiliated Cancer Hospital of Inner Mongolia Medical University, Hohhot, Inner Mongolia, China; 3 School of Pharmacy, Inner Mongolia Medical University, Hohhot, Inner Mongolia, China; 4 Pathology Department, Peking University Cancer Hospital/Affiliated Cancer Hospital of Inner Mongolia Medical University, Hohhot, Inner Mongolia, China; 5 Department of Medical Simulated Center, Inner Mongolia Medical University, Hohhot, Inner Mongolia, China; 6 Key Laboratory of Carcinogenesis and Translational Research (Ministry of Education), Department of Pathology, Peking University Cancer Hospital & Institute, Beijing, China

**Keywords:** cancer treatment-induced thrombocytopenia, machine learning, predictive models, recombinant human thrombopoietin, stacking ensemble mode

## Abstract

**Background:**

Cancer treatment-induced thrombocytopenia (CTIT) is a common adverse effect of cancer therapy. CTIT increases the risk of bleeding, prolongs hospital stays, raises medical costs, and can negatively impact anti-tumor treatment outcomes, potentially leading to patient death. Therefore, it is crucial to initiate platelet-boosting therapy in a timely manner based on the individual circumstances of patients experiencing CTIT.

**Methods:**

Patients who developed cancer treatment-induced thrombocytopenia and received Rh-TPO treatment from January 2023 and December 2023 were obtained for establishing the dataset. With absolute platelets increase as the outcome variable, univariate analysis was performed to screen out statistically significant factors, and 18 clinical variables were selected as initial features. The least absolute shrinkage and selection operator (LASSO) regression analysis was then used to identify the most important features. Based on this, a stacking ensemble model was constructed using cross-validation with out-of-fold predictions to prevent information leakage, and the predictive performance of the model was evaluated. Finally, the SHapley Additive exPlanations (SHAP) algorithm was used to explain the model, and a visual analysis of the features was conducted.

**Results:**

A total of 400 inpatients who developed cancer treatment-induced thrombocytopenia and received Rh-TPO treatment were included, of which 280 inpatients were assigned to the training set and 1,20 to the testing set. After LASSO regression screening, the study identified 7 key features: ethnicity, height, baseline serum creatinine, pre-chemotherapy platelet count, follow-up days after chemotherapy, platelet count before Rh-TPO, and duration of Rh-TPO treatment. We compared the performance of different machine learning models and selected the best three models as base models. Combined with Linear Regression as the meta-learner, we built a stacking ensemble model using 3-fold cross-validation with out-of-fold predictions. The stacking ensemble model showed best prediction ability compared to independent models with R² of 0.77 (training) and 0.74 (testing), MAE of 6.39 (training) and 8.34 (testing), MSE of 62.17 (training) and 97.70 (testing), RMSE of 7.88 (training) and 9.88 (testing), and MAPE of 0.09 (training) and 0.12 (testing). SHAP analysis showed that pre-chemotherapy PLT value and the follow-up days after chemotherapy were the important features affecting the prediction results.

**Conclusion:**

The predictive model developed in this study could be beneficial for accurately predicting the improvement in platelet count in patients with CTIT who use Rh-TPO, facilitating timely assistance for patients in avoiding the risks caused by a drop in platelet count in a timely manner.

## 1 Background

Cancer Treatment-Induced Thrombocytopenia (CTIT) is a common adverse reaction associated with anti-cancer drugs, affecting up to 15% to 25% of patients undergoing anti-tumor therapy (1, 2), with grade III/IV cases occurring at a rate of approximately 3% (3). CTIT leads to severe platelet reduction, increasing the risk of bleeding, prolonging hospital stays, raising medical costs, and in severe cases, leading to alterations in chemotherapy regimens, dose reductions, or even discontinuation of treatment, which negatively impacts the outcomes of anti-tumor therapy and potentially lead to patient mortality (4). To reduce or prevent the survival risks posed by CTIT, various domestic and international guidelines and consensus statements, in conjunction with the evolving landscape of anti-tumor and platelet-boosting therapeutic agents, have refined the standard prevention and treatment strategies for CTIT and established corresponding standards (5–7). Among thrombopoietic agents, Recombinant Human Thrombopoietin (Rh-TPO) is one of the most commonly used treatments in China to prevent and treat CTIT, significantly enhancing platelet recovery (8, 9). However, despite these guidelines and recommendations, several challenges and uncertainties remain in clinical practice, including individual patient variability, regional differences, cancer types, and treatment regimens, all of which can significantly influence the effectiveness of Rh-TPO in treating CTIT.

Therefore, developing an effective predictive model to forecast the platelet counts improvement in patients with CTIT before initiating Rh-TPO treatment is crucial for achieving personalized treatment, assisting clinical decision-making, and identifying patients who are more likely to respond to treatment, thereby optimizing the allocation of medical resources and improving treatment outcomes.

Recent studies have demonstrated that integrative and bioinformatics-driven approaches enable systematic characterization of complex molecular interactions and robust identification of candidate biomarkers across heterogeneous clinical conditions. These findings highlight the inherently high-dimensional and nonlinear nature of disease pathophysiology-features that fundamentally limit the capacity of conventional statistical methods to capture underlying biological complexity. As a result, sophisticated data-driven methodologies—particularly machine learning—are increasingly indispensable for modeling such intricate relationships and enhancing the accuracy, generalizability, and clinical utility of predictive models (10–13). Machine learning methods are a promising data statistical analysis technique in the field of medicine and an essential component of artificial intelligence. Compared with traditional statistical methods, machine learning can mine large datasets for fitting, enhance data processing capabilities, they offer significant advantages in handling high-dimensional, large-sample data (14–17). Various machine learning methods have been applied to drug efficacy prediction, including Support Vector Machine, Random Forest, XGBoost, and LightGBM (18–21). Ensemble learning methods, which combine multiple base learners, have garnered significant attention due to their ability to effectively enhance predictive performance. Stacking is one of the most popular ensemble algorithms and has demonstrated strong predictive performance in various biomedical scenarios (22, 23). Nonetheless, there are no effective measures to predict the efficacy of Rh-TPO in the treatment of CTIT.

In this study, we collected the clinical data of 400 cancer patients with CTIT, used statistical analysis to screen variables, used LASSO regression analysis to screen clinical features, and compared the performance of different machine learning algorithms. Based on the model performance ranking, we established a stacking ensemble model, which used the three best-performing models as the base models and performed linear regression combination as the meta-model. At the same time, the SHAP method was used to explain the features, and the clinical application value of the model was analyzed.

## 2 Methods

### 2.1 Data sources and study population

This retrospective study included the cases of patients who developed CTIT and received Rh-TPO treatment from January 2023 to December 2023. Patients under the age of 18, incomplete case records, and those with thrombocytopenia caused by other diseases were excluded. Patient consent was waived due to the retrospective nature of the study. We collected demographic information (gender, ethnicity, age, height, weight), the platelet counts of patients prior to cancer therapy, days of post cancer therapy follow-up, and the platelet counts before Rh-TPO administration, treatment information (the dosage and duration of Rh-TPO administration, the platelet counts recorded after completing Rh-TPO treatment), and laboratory values including baseline alanine aminotransferase (ALT), baseline aspartate aminotransferase (AST), baseline serum creatinine, baseline hemoglobin concentration.

The primary outcome measure was the platelet counts after completing Rh-TPO treatment. The rationale for this approach was based on several variables: platelet counts before cancer therapy, days of post-chemotherapy rechecks (the follow-up days after chemotherapy, representing the time from chemotherapy initiation to the first blood routine examination), the platelet counts before Rh-TPO treatment, and the duration of Rh-TPO treatment (the number of days of Rh-TPO therapy, determined by the treating physician based on clinical guidelines and patient response). The ethnicity variable was coded as Han = 0, Meng = 1, Hui = 2, Man = 3, and others = 4.

### 2.2 Data preprocessing and features selection

In this study, we employed the least absolute shrinkage and selection operator (LASSO) model regression to identify the most significant features for our predictive model. This approach not only reduces the risk of overfitting but also enhances the model’s generalization. After univariate analysis, 18 statistically significant variables (*P < 0.05*) were included in LASSO regression. We used LassoCV with 10-fold cross-validation to determine the optimal lambda (λ) value. The dataset was randomly divided into training set (n=280, 70%) and testing set (n=120, 30%).

### 2.3 Independent ML model development

We constructed six independent machine learning models: Support Vector Machine Regressor (SVM), Random Forest Regressor (RF), Light Gradient Boosting Machine (LightGBM), Extreme Gradient Boosting Regressor (XGBoost), Categorical Boosting Regressor (CatBoost), and Categorical Boosting with Genetic Algorithm Regressor (CatBoost-GA), with each trained independently for the prediction task. Model performance was evaluated using coefficient of determination (R²), mean absolute error (MAE), mean square error (MSE), root mean square error (RMSE), and mean absolute percentage error (MAPE).

### 2.4 Stacking ensemble learning

To enhance the accuracy and generalization ability of the final prediction model, this study employed a stacking algorithm to integrate multiple base models, thereby improving prediction accuracy. The stacking ensemble was implemented using scikit-learn’s StackingRegressor with 3-fold cross-validation (cv=3). This implementation uses out-of-fold (OOF) predictions to generate meta-features, which prevents information leakage between the base models and the meta-learner. Specifically, for each fold, the base models are trained on the training portion and make predictions on the held-out portion. These OOF predictions are then used to train the meta-learner, ensuring that the meta-learner never sees predictions made on data that was used to train the base models.

**Figure 1:**
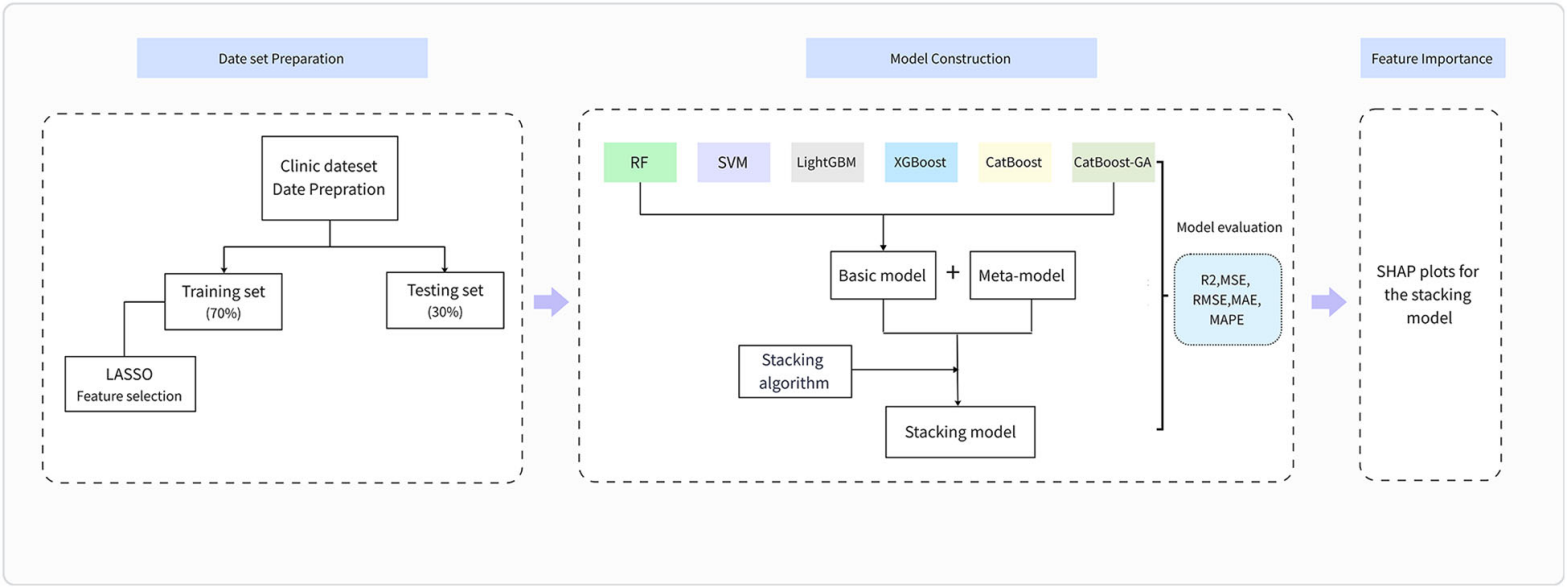
The modeling process of this study. LASSO, the least absolute shrinkage and selection operator; XGBoost, eXtreme gradient boosting; LightGBM, light gradient boosting machine; RF, random forest regressor; SVM, support vector machine regressor; CatBoost, categorical boosting regressor; CatBoost-GA, categorical boosting with genetic algorithm regressor; MSE, mean squared error; MAE, mean absolute error; RMSE, root mean square error; MAPE, mean absolute percentage error.

The hyperparameters for each base model in the stacking ensemble were configured as follows: Random Forest (n_estimators=100, random_state=42), XGBoost (n_estimators=100, random_state=42, verbosity=0), and CatBoost (n_estimators=100, random_state=42, verbose=False). Based on performance evaluation, RF, XGBoost, and CatBoost were selected as base learners, with Linear Regression as the meta-learner. The modeling process of this study was shown in **Figure 1**.

### 2.5 Model interpretation

This study employs the SHapley Additive exPlanations (SHAP)method to interpret model results, presenting both SHAP summary plots and average SHAP values plots. In the SHAP summary plot, the Y-axis represents features, and the X-axis indicates the SHAP values. Each point represents a sample, with the color gradient indicating the feature values. By observing the distribution of each feature, one can understand the relationship between features and model predictions. In the average SHAP values plot, the Y-axis also represents features, while the X-axis represents the average absolute SHAP value, reflecting the overall importance of each feature in model predictions.

### 2.6 Statistical analysis

Machine learning model training and testing were conducted in the Python 3.7.4 environment, using Scikit-learn (version 1.0.2) and SHAP (version 0.40.0). Statistical analysis of clinical data was performed using SPSS 27.0. Continuous variables were expressed as mean ± standard deviation (SD) when the data were normally distributed; for non-normally distributed data, continuous variables were expressed as median with first and third quartiles [M(P25, P75)]. Categorical data were presented as the number of cases and percentages (%).

## 3 Results

### 3.1 Baseline characteristics

A total of 400 cases had been included in the study, with patient data covering age, gender, ethnicity, height, weight, platelet counts before cancer therapy (PLTs before cancer therapy), days of post cancer therapy follow-up, platelet counts before Rh-TPO administration(PLTs before Rh-TPO), platelet counts after Rh-TPO administration (PLTs after Rh-TPO), duration of Rh-TPO, as well as the baseline alanine aminotransferase (ALT), aspartate aminotransferase (AST), serum creatinine, hemoglobin levels. These were all used as features for model building. As shown in **Table 1**, the study found that among the 400 tumor patients with CTIT, the majority were elderly women over 60 years old, Han ethnicity predominated, though patients from other ethnic minorities also accounted for a certain proportion. Before anti-tumor treatment, platelet counts were within normal range; median pre-Rh-TPO platelet count was 59.8×10^9^/L. The median Rh-TPO treatment duration was 5 days.

**Table 1:** The baseline characteristics of 400 cases.

Variables	N	Value
Age, years, M (P_25_, P_75_)	400	62 (54.2, 68)
Height, cm, M (P_25_, P_75_)	400	165 (160, 173)
Weight, kg, M (P_25_, P_75_)	400	60 (55, 68)
Baseline ALT, u/l, M (P_25_, P_75_)	400	19 (12, 28)
Baseline AST, u/l, M(P_25_, P_75_)	400	21 (15, 30.8)
Baseline Serum Creatinine, umol/l u/l, M (P_25_,P_75_)	400	66 (55, 78)
PLTs before cancer therapy, 10^9^/l, M (P_25_, P_75_)	400	160 (120, 202.5)
Days of post cancer therapy follow-up, M (P_25_, P_75_)	400	11 (7, 17)
Duration of Rh-TPO,u/l,M (P_25_,P_75_), 10^9^/l, M (P_25_, P_75_)	400	5 (4, 7)
PLTs after Rh-TPO, 10^9^/l, M (P_25_, P_75_)	400	78 (52, 98)
PLTs before Rh-TPO,1 0^9^/l, Mean ± SD	400	55.8 ± 21.2
Baseline Hemoglobin, g/l, Mean ± SD	400	115.0 ± 26.2
Gender, n (%)		
Male	197	49.3%
Female	203	50.7%
Ethnicity, n (%)		
Han	367	91.8%
Mongolian	26	6.5%
Hui	5	1.3%
Manchu	2	0.4%

### 3.2 Lasso regression for feature variable selection

**Figure 2:**
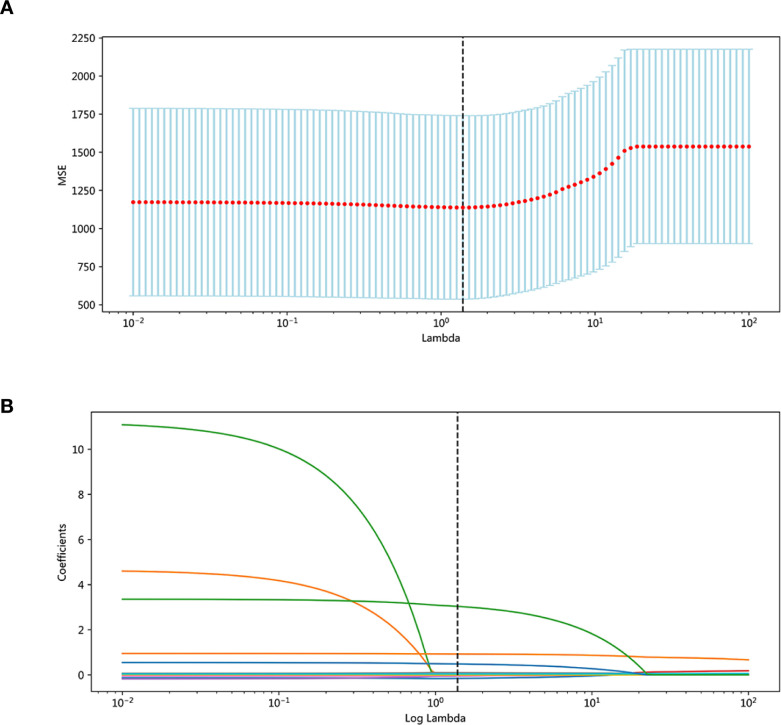
**(A)** LASSO cross-validation: MSE vs log(λ). **(B)** LASSO coefficient paths.

The dataset contained no missing data, eliminating the need for imputation. The results of LASSO regression on feature screening were shown in **Figure 2**. We identified seven feature variables with non-zero coefficients from the training set, specifically: ethnicity, height (cm), baseline creatinine, platelet counts before cancer therapy, days of post cancer therapy follow-up, platelet counts before Rh-TPO administration (PLTs before Rh-TPO), and duration of Rh-TPO treatment. These features were then used for subsequent model construction.

### 3.3 Model evaluation and performance

**Figure 3:**
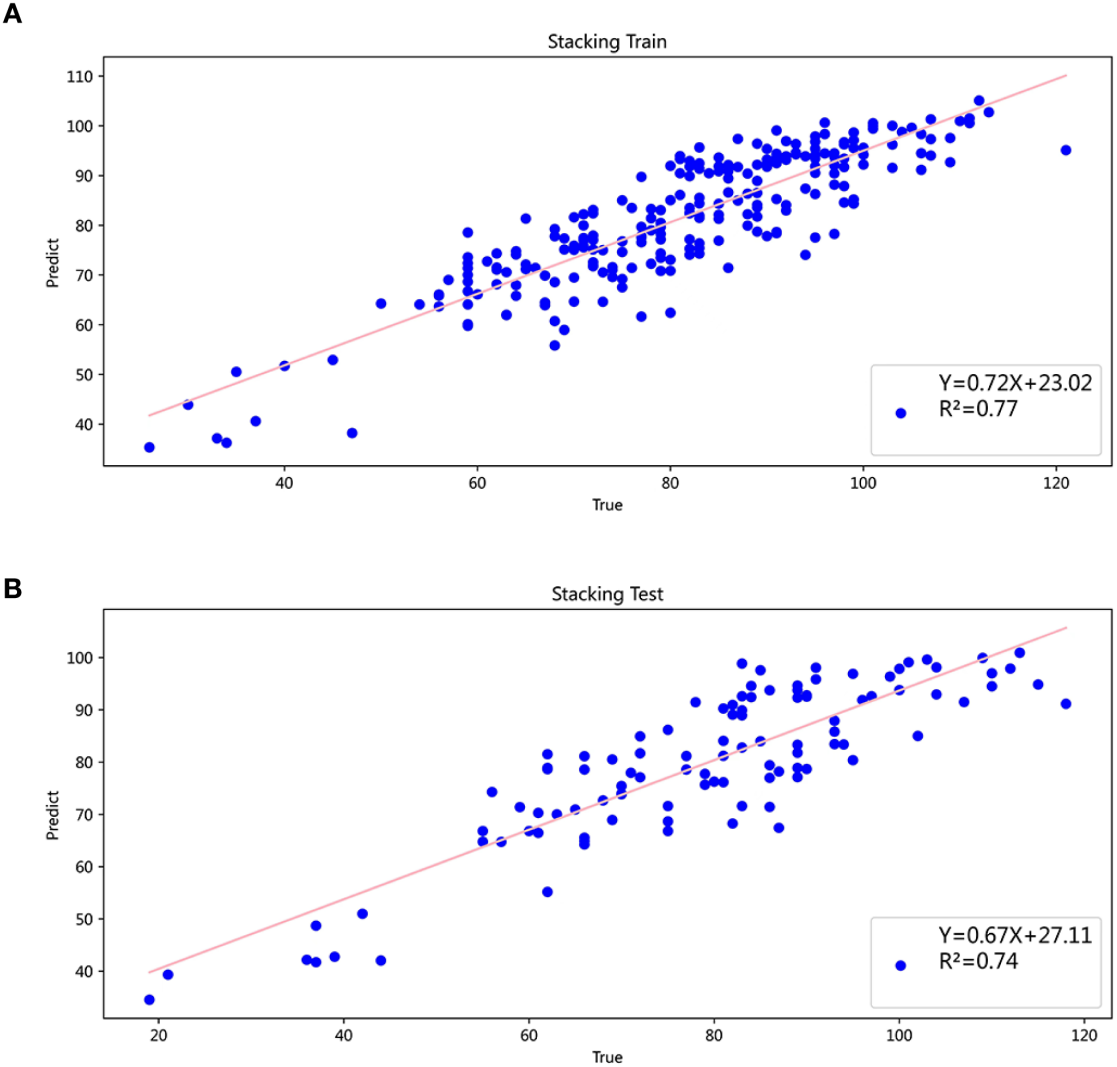
**(A)** Stacking model predictions vs actual values (training set). **(B)** Stacking model predictions vs actual values (testing set).

**Figure 4:**
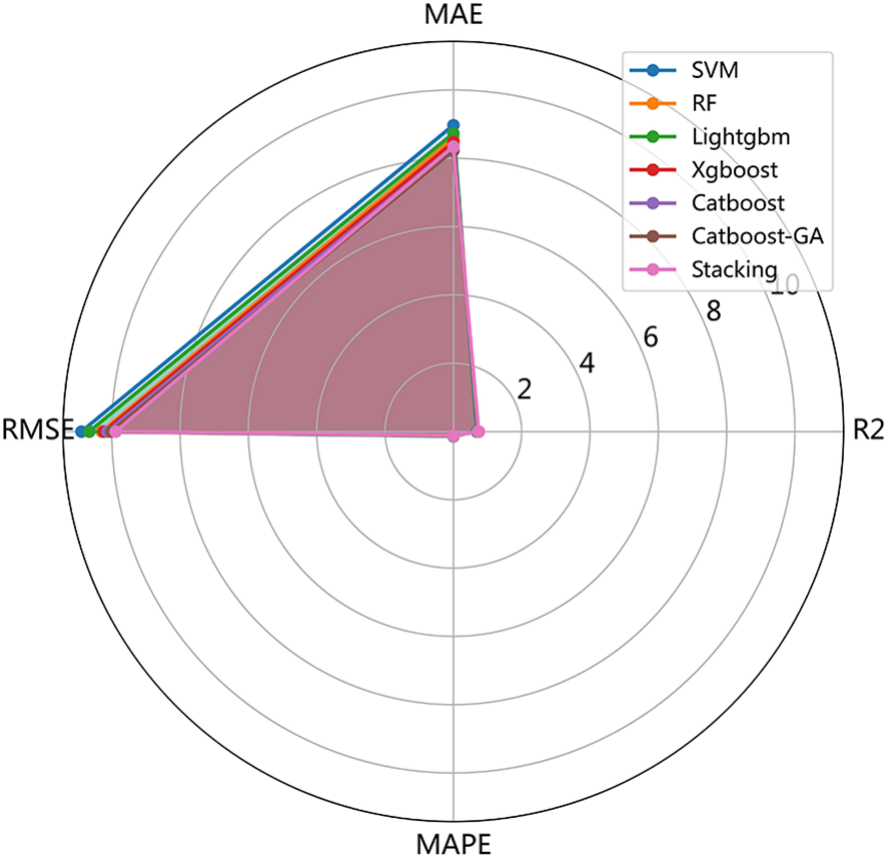
The radar chart for comprehensive comparison of 7 models ability of the stacking ensemble model on the testing set.

RF, XGB, and CatBoost exhibited the best performance among the six independent models. Therefore, we selected these three models as base models and used Linear Regression as the meta-model for the stacking learning. The prediction performance of the six independent models and the stacking ensemble model is presented in **Table 2**. The stacking ensemble model showed the best prediction ability compared to independent models with R² of 0.77 (training) and 0.74 (testing). The predictive ability of the stacking ensemble model on the training and testing set is illustrated in **Figure 3**. A comparison of the comprehensive performance of each model is shown in **Figure 4**.

**Table 2:** The predictive performance of seven models.

Models	Dataset	R^2^	MAE	MSE	RMSE	MAPE
RF	Training set	0.77	6.36	62.30	7.89	0.09
	Testing set	0.72	8.60	106.00	10.30	0.13
SVM	Training set	0.64	7.5	97.17	9.86	0.10
	Testing set	0.69	8.97	118.52	10.89	0.13
XGBoost	Training set	0.75	6.62	66.63	8.16	0.09
	Testing set	0.72	8.48	104.75	10.23	0.13
LightGBM	Training set	0.72	6.94	74.53	8.63	0.10
	Testing set	0.70	8.73	113.63	10.66	0.14
CatBoost	Training set	0.77	6.32	61.40	7.84	0.09
	Testing set	0.73	8.35	102.50	10.12	0.13
CatBoost-GA	Training set	0.78	6.25	59.42	7.71	0.09
	Testing set	0.73	8.23	100.45	10.02	0.12
Stacking Ensemble	Training set	0.77	6.39	62.17	7.88	0.09
	Testing set	0.74	8.34	97.70	9.88	0.12

### 3.4 Model interpretation

**Figure 5:**
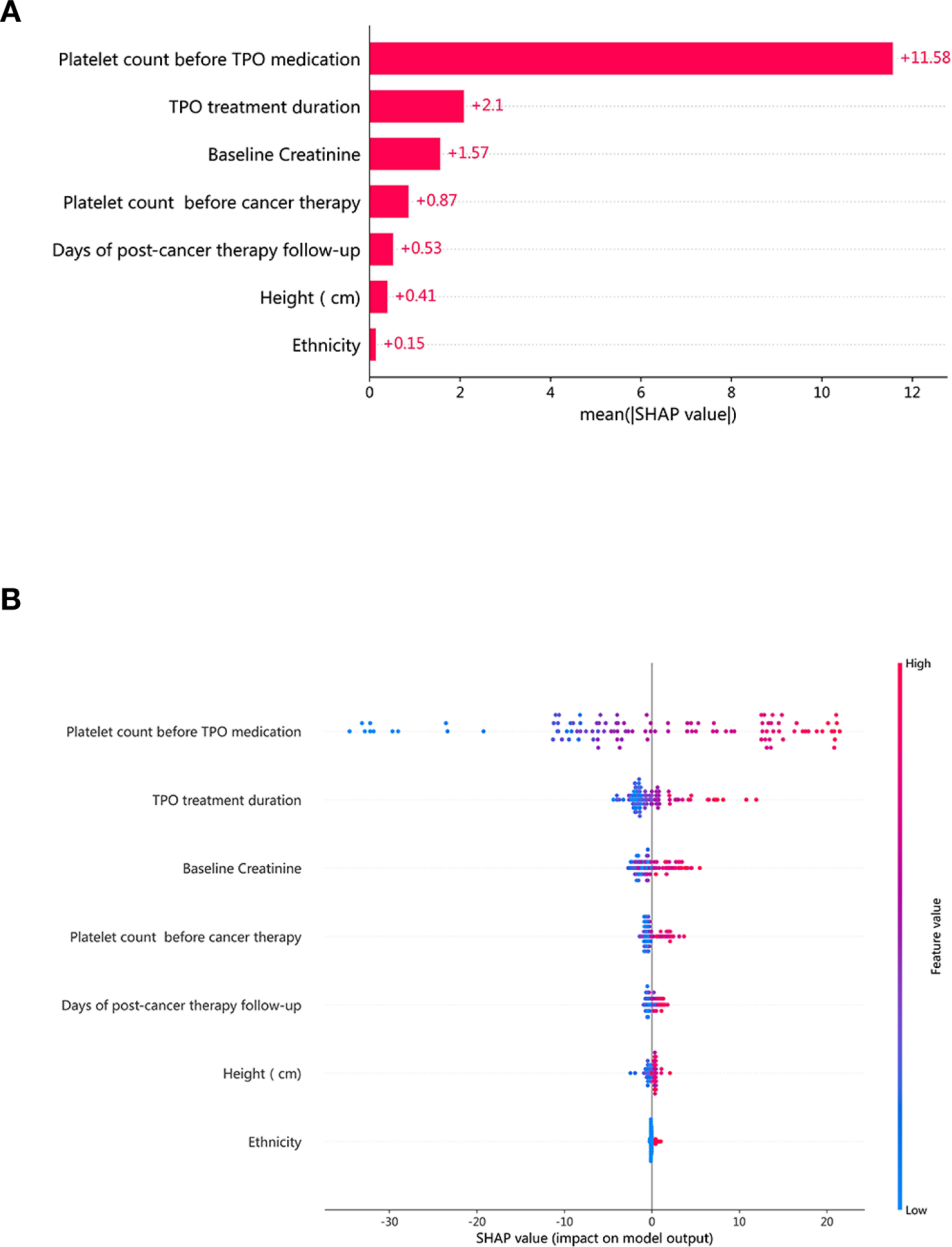
**(A)** The ranking of the average SHAP values of the 7 features in the stacking model. **(B)** SHAP summary plot showing feature effects.

After model evaluation, the stacking ensemble model was finally selected as the optimal model. The feature importance of the optimal model was ranked based on SHAP values. **Figure 5A** shows the average SHAP values for each feature in the stacking ensemble model. Pre-chemotherapy PLT value and follow-up days after chemotherapy emerged as the top features contributing to model predictions. **Figure 5B** displays the SHAP summary plot, where each point represents a sample. The color indicates feature value (red for high, blue for low), and position shows impact on prediction. These visualizations provide interpretable insights into the model’s decision-making process.

### 3.5 Visual interface

To achieve real-time prediction of Rh-TPO treatment effects, we developed an interactive web application using Streamlit. The website address is https://platelet-qigobpmrnyevbrnyfkcftm.streamlit.app/. Users can input specific patient information including ethnicity, height, baseline serum creatinine, pre-chemotherapy platelet count, follow-up days after chemotherapy, platelet count before Rh-TPO treatment, and duration of Rh-TPO treatment. The system provides real-time predictions using the trained stacking ensemble model.

## 4 Discussion

In recent years, remarkable progress has been made in the application of machine learning models in the prediction of the efficacy of antineoplastic drugs. Among the many prediction models, Ensemble Learning method is particularly attractive, especially the Stacking method, because it can effectively combine the advantages of different models to improve the prediction performance, and is widely used in a variety of cancer therapy-related tasks. In this study, the research subjects is the platelet counts following Rh-TPO treatment, which is a continuous variable. The objective of the predictive model was to minimize the discrepancy between predicted and actual measured values, thereby classifying its performance as a regression problem. Consequently, we selected R², MSE, MAE as the evaluation metrics for model performance. It is widely acknowledged in the literature that a model demonstrates optimal predictive performance when the R^2^ value for the training set ranges from 0.8 to 1 and the R^2^ value for the validation set lies between 0.5 and 1, while minimizing MSE and MAE further enhances model accuracy. Despite the relatively small test set of only 120 cases, the stacking ensemble model achieved R² values exceeding 0.7 on the test set, demonstrating robust fitting performance. Compared to six independent models, the stacking ensemble model exhibited the highest R² value and the lowest error. This indicates that the application of machine learning, particularly stacking methods, not only enhances the accuracy of tumor drug response prediction but also highlights the advantages of stacking in addressing complex problems. Unlike traditional single models, the stacking method improves overall prediction performance by weighting and adjusting the outputs of multiple base models. Based on our experimental results, the stacking method significantly enhanced the accuracy of drug response prediction while reducing the model’s sensitivity to individual features or data noise (20). When selecting base models, XGBoost and CatBoost emerge as two of the most prominent gradient boosting tree (GBDT) algorithms in contemporary machine learning. XGBoost excels in handling non-linear data, capturing feature interactions, processing missing values, and implementing model regularization (21). In contrast, CatBoost demonstrates exceptional proficiency in managing categorical variables through its innovative sorting method and efficient encoding techniques, enabling it to perform remarkably well across a variety of complex real-world problems (22). By leveraging these two models as base learners, we can fully capitalize on their respective strengths for diverse tasks. Despite its relative simplicity, linear regression is employed as a meta-learner in stacking methods. Its advantages include reducing overfitting and enhancing generalization capabilities, particularly when aggregating predictions from multiple base models; these benefits are even more pronounced in such contexts. In this research, we discovered that employing linear regression as a meta-learner to combine the outputs of multiple base models successfully mitigated the bias and variance issues of each model, leading to notable improvements in both prediction stability and accuracy. The inherent simplicity and interpretability of linear regression provided us with valuable insights into how different base models contribute to the final prediction outcomes. When compared to more complex deep learning models or other highly nonlinear meta-learners, the performance of linear regression in this context was surprisingly effective. We propose that the role of linear regression within stacking ensemble models goes beyond simply acting as a weighting mechanism. Instead, it fosters collaborative interactions among the predictions from various base models by leveraging learned relationships, which helps to reduce potential errors associated with individual models. This approach allows for a more robust and accurate predictive framework, enhancing the overall reliability of the ensemble method (23–25).

In clinical settings, the balance between predictive performance and interpretability must be carefully considered when applying models. Therefore, interpreting feature contributions is particularly important. Based on SHAP, we conducted multiple analyses and final ranking of feature importance. Among these features, the platelet counts before Rh-TPO treatment ranked highest, with its average SHAP value significantly higher than the other six features, indicating its greatest contribution to the prediction model. This finding indirectly suggests that patients with higher pre-treatment platelet counts may experience better responses to Rh-TPO in improving platelet counts associated with CTIT. Several published clinical studies support this idea, showing that patients with higher initial platelet counts recover more quickly after Rh-TPO treatment and have a lower risk of severe bleeding events (26–29). Rh-TPO accelerates platelet production by promoting the proliferation and maturation of megakaryocytes, a process based on the hematopoietic potential of the patient’s own bone marrow. Low platelet counts prior to Rh-TPO treatment typically indicate a higher risk of bleeding, which can prolong the treatment duration and slow down platelet recovery. Additionally, factors such as a history of pelvic radiotherapy or chronic liver disease can further impact the efficacy and duration of Rh-TPO treatment. Therefore, understanding a patient’s pre-treatment platelet counts can assist physicians in making personalized treatment adjustments when utilizing Rh-TPO. During anti-tumor drug therapy, clinicians and clinical pharmacists should conduct a comprehensive evaluation of CTIT risk, considering the patient’s medical history and previous treatments, while regularly monitoring platelet counts. When platelet counts fall to the lower limit of normal values, a thorough assessment is warranted; patients identified as at risk for bleeding should promptly initiate Rh-TPO therapy. The second most significant characteristic associated with SHAP values is the duration of Rh-TPO treatment. Prolonged administration of Rh-TPO has been correlated with more favorable improvements in patients’ platelet counts. Rh-TPO promotes the final production and release of platelets by stimulating megakaryocytes in the bone marrow, a process that typically takes several days to weeks and varies among individuals. Consequently, the therapeutic effect of Rh-TPO is not immediate, and the duration of treatment directly influences the timeline for observing therapeutic benefits. If the treatment cycle is brief, patients may not fully capitalize on the effects of Rh-TPO, resulting in only modest or transient improvements in platelet levels. A longer treatment duration often allows more time for the drug to exert its effects, thereby facilitating ongoing platelet production and enabling patient platelet counts to gradually return to within normal ranges. Especially for patients with thrombocytopenia caused by chemotherapy, bone marrow suppression, or other underlying conditions, the rate of platelet consumption is significantly increased. Short-term Rh-TPO treatment may only address immediate platelet deficiency needs, whereas a prolonged course can help maintain stable platelet levels and reduce complications associated with thrombocytopenia, such as bleeding. In cases of chronic or recurrent thrombocytopenia, the duration of Rh-TPO therapy directly influences the speed and extent of platelet recovery. Therefore, extending the treatment period may be particularly beneficial for patients with persistent or recurrent conditions. However, it is important to note that long-term use of Rh-TPO may increase the risk of thrombosis and induce immune system changes (30). Consequently, the treatment regimen should be individualized based on the patient’s specific clinical condition. While a longer treatment course can enhance therapeutic efficacy, careful consideration must be given to balancing risks and benefits.

This study has several limitations. Firstly, the dataset used is retrospective in nature, which may limit the model’s generalization ability. Future research should aim to enhance the model’s generalizability by incorporating more diverse and prospective data sources. Secondly, the amount of data included in this study is relatively limited. Expanding the dataset is crucial for increasing the frequency of machine learning iterations and enhancing data processing capabilities, thereby further optimizing the model’s accuracy. Thirdly, the moderate R² value (0.74) on the testing set indicates that a substantial portion of variance in treatment response remains unexplained, suggesting that additional predictive factors exist that were not captured in our current feature set. Fourthly, the inclusion of Rh-TPO treatment duration as a predictor warrants careful interpretation: while this variable was pre-specified based on CSCO clinical guidelines (5–14 days), its potential temporal overlap with outcome measurement means the model is best suited for treatment response assessment rather than pure “prediction at treatment initiation” scenarios. For true pre-treatment prediction, future models could exclude treatment duration and focus solely on baseline variables. Fifthly, the follow-up days after chemotherapy variable represents the timing of blood examination rather than a treatment decision, and its relationship with outcomes requires careful interpretation in clinical application. Finally, while we used cross-validation to prevent overfitting, prospective external validation studies are needed to confirm the clinical utility of our model. In future work, we plan to explore integrating more complex meta-learners (such as deep learning models) with traditional linear regression. Additionally, the Streamlit-based web application developed in this study serves only as a proof-of-concept tool. Before being implemented in real-world clinical settings, the model would require comprehensive external validation, periodic recalibration, and continuous monitoring for potential data drift. By leveraging advanced feature engineering and hyperparameter optimization techniques, we aim to further improve the performance of the stacking method.

## 5 Conclusion

The findings of this study indicate that the stacking ensemble model demonstrates commendable performance in predicting the efficacy of Rh-TPO treatment for chemotherapy-induced thrombocytopenia (CTIT). By effectively integrating multiple machine learning algorithms, the model provides accurate predictions for platelet count improvement, offering a reference for clinical decision-making and aiding in the optimization of personalized treatment strategies for CTIT patients.

## Data Availability

The original contributions presented in the study are included in the article/supplementary material. Further inquiries can be directed to the corresponding authors.
